# Lipids and Lipid-Mediated Signaling in Plant–Pathogen Interactions

**DOI:** 10.3390/ijms25137255

**Published:** 2024-07-01

**Authors:** Elżbieta Kuźniak, Ewa Gajewska

**Affiliations:** Department of Plant Physiology and Biochemistry, University of Lodz, 90-237 Łódź, Poland; ewa.gajewska@biol.uni.lodz.pl

**Keywords:** biotic stress, lipids, lipid signaling, oxylipins, plant immunity, plant–pathogen interaction, surface lipids

## Abstract

Plant lipids are essential cell constituents with many structural, storage, signaling, and defensive functions. During plant–pathogen interactions, lipids play parts in both the preexisting passive defense mechanisms and the pathogen-induced immune responses at the local and systemic levels. They interact with various components of the plant immune network and can modulate plant defense both positively and negatively. Under biotic stress, lipid signaling is mostly associated with oxygenated natural products derived from unsaturated fatty acids, known as oxylipins; among these, jasmonic acid has been of great interest as a specific mediator of plant defense against necrotrophic pathogens. Although numerous studies have documented the contribution of oxylipins and other lipid-derived species in plant immunity, their specific roles in plant–pathogen interactions and their involvement in the signaling network require further elucidation. This review presents the most relevant and recent studies on lipids and lipid-derived signaling molecules involved in plant–pathogen interactions, with the aim of providing a deeper insight into the mechanisms underpinning lipid-mediated regulation of the plant immune system.

## 1. Introduction

Lipids and the products of lipid metabolism play essential roles in the defense mechanisms of plants. Biotic stress alters the metabolism of lipids, which have both passive and active functions in plant immunity. Lipids are the main structural components of the cuticle and plasma membrane, constituting the first physical barriers that the pathogens encounter. Some lipid and lipid-derived species exhibit direct antimicrobial activity. They also act as a source of signals perceived by both the invading pathogen and the plant, which regulate plant–pathogen interactions [1,2]. While the best-recognized lipid-derived signals in plants are oxylipins, including jasmonic acid (JA) [3], many other lipid derivatives involved in plant defense signaling have now been identified, and their mechanisms of action are under active investigation [4]. 

In pathogen-challenged plants, dynamic changes in the lipid pool can trigger localized responses at the sites of pathogen entry, and lipid signals can also be transmitted systemically throughout the plant [2]. Some lipid metabolism alterations also occur in the invading pathogens, and there is increasing evidence that lipids are part of the chemical language of communication between plants and microorganisms [5]. Although intense recent studies have expanded our knowledge of the roles of lipids in plant–pathogen interactions, there is still a need to further understand how lipids and lipid-derived signals can modulate plant defense. From a broader perspective, a detailed knowledge of the roles of lipids in plant–pathogen interactions may allow the identification of new strategies for plant disease management.

This review examines the numerous roles of lipids and lipid-derived signals in modulating plant–pathogen interactions, with a particular focus on plant colonization and the signaling of the downstream defense responses. It describes the main classes of plant lipids and discusses how post-infectious changes in the lipid pool contribute to local and systemic defense responses in the plant.

## 2. Classification, Composition, and Function of Plant Lipids

Plant lipids can be classified as glycerolipids, sphingolipids, sterols, polyketides, and prenol lipids based on their chemical structure. Of these, the most abundant are the glycerolipids, comprising phospholipids (PLs), galactolipids (GLs), triacylglycerols (TAGs), and sulpholipids (SLs) [6,7]. 

Phospholipids are composed of diacylglycerol (DAG), i.e., two fatty acids (FAs) esterified to the sn-1 and sn-2 positions of a glycerol backbone, and an alcohol-modified phosphate head group attached to the sn-3 position. Depending on the alcohol moiety present in the head group, plant PLs are divided into phosphatidylcholine (PC), phosphatidylethanolamine (PE), phosphatidylglycerol (PG), phosphatidylserine (PS), phosphatidylinositol (PI) and phosphatidic acid (PA); the latter carries only a phosphate as the polar head group. Galactolipids are represented by monogalactosyldiacylglycerol (MGDG) and digalactosyldiacylglycerol (DGDG), which contain DAG and one or two galactose molecules attached to the sn-3 position of the glycerol backbone, respectively. Triacylglycerol (TAG) comprises a glycerol backbone bearing three esterified FAs. SLs are characterized by the presence of a sulfur-containing functional group. In plants, they are represented mainly by sulphoquinovosyldiacylglycerol (SQDG), an anionic glycolipid composed of sulphoquinovose (6-deoxy-6-sulphoglucose) linked to the sn-3 position of the glycerol backbone in DAG [8,9].

Sphingolipids comprise a ceramide backbone composed of an amino alcohol (long chain base, LCB) linked to FAs with an amide bond. Ceramide structure can be further modified by adding glycosyl residues and other polar phosphate-containing head groups. Plant sphingolipids can be divided into four main classes, namely, ceramides (Cers), glycosylceramides (GCers), glycosyl inositolphosphoceramides (GIPCs), and free LCBs [10,11].

Plant sterols (phytosterols) are isoprenoids formed by a cylcopentaperhydrophenantren moiety; this is a structure made up of four rigid rings which is hydroxylated at position 3. Phytosterols can occur in free forms or conjugated ones such as sterol glucosides (SGs) and acylated sterol glucosides (ASGs). The major phytosterols are stigmasterol, β-sitosterol, and campesterol [12]. Polyketides are polymers composed of acyl residues synthesized by polyketide synthases. Prenol lipids (terpenoids) are synthesized from the 5-carbon precursors, namely, isopentenyl diphosphate and dimethyl diphosphate, which are mainly produced via the mevalonic acid pathway. They contain one or more isoprene units in their structure [13].

FAs can exist as free molecules and as important components of lipids. A large number are known to exist, which differ in their chain length and degree of saturation. FAs can be classified as short-chain (up to 5 or even 7 carbons), medium-chain (6–8 up to 12–14 carbons), long-chain (13–18 up to 22 carbons), or very-long-chain fatty acids (VLCFAs, longer than 22 carbons) [6]. The aliphatic chain can be saturated, i.e., without double bonds, or unsaturated, i.e., with at least one double bond. In plants, FAs with 16 carbon atoms represent about 30% of total FAs, and those with 18 carbons about 70%. The most abundant FA species are linoleic acid (C18:2) and α-linolenic acid (C18:3), representing about 80% of 18C and nearly 55% of total FAs [14]. Considerable amounts of hexadecatrienoic acid (C16:3) are present in the leaves of *Arabidopsis thaliana* and other 16:3 plants, where they represent about 14% of the total FA content [15].

**Figure 1:**
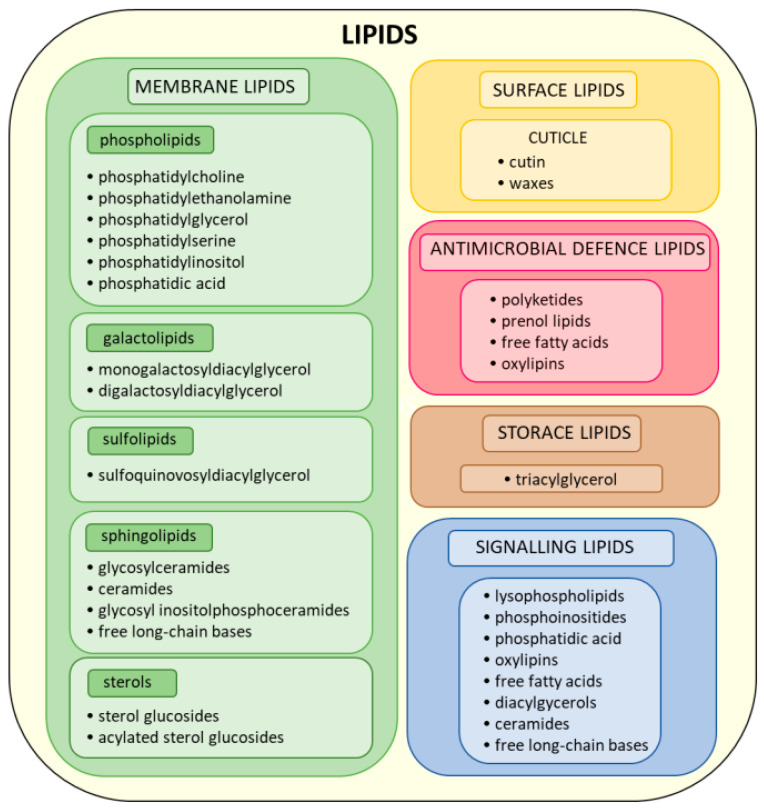
Chemical structure- and function-based classification of plant lipids.

In plants, lipids perform various structural, storage, signaling, and defensive functions, among others. They are the main structural components of plant membranes and the cuticle covering the plant surface (Figure 1). Phospholipids, galactolipids, sulpholipids, sphingolipids, and sterols serve as key constituents of plant membranes. Particular types of plant membranes differ in their lipid composition. The total pool of plasma membrane lipids is composed of phospholipids (30–50%), sphingolipids (5–40%), and sterols (20–50%). The main phospholipids present in plasma membrane are PC (25–45%) and PE (30–40%), and the less abundant classes are PS (3–12%), PG (2–15%), PI (2–11%), and PA (0–20%) [12,16]. In contrast to the plasma membrane and other extraplastidial membranes, chloroplast membranes are characterized by a high amount of galactolipids. Thylakoids and the inner chloroplast envelope contain approximately 49% MGDG, 30% DGDG, 5% SQDG, and 8% PG. The outer envelope is composed of 17% MGDG, 29% DGDG, 6% SQDG, 10% PG, and 32% PC [9]. 

Plant surface lipids, including cutin and cuticular waxes, are components of the cuticle, a continuous hydrophobic layer covering the epidermis. Cutin is the core structural polymer, and it is composed of hydroxy and epoxy C16 and C18 FAs and glycerol. Cuticular waxes are a mixture of mostly aliphatic 20C to 40C VLCFAs derivatives and variable amounts of triterpenoids and phenylpropanoids [17]. 

TAG is the primary storage lipid in plants. It can be found within cytoplasmic lipid droplets in seeds and chloroplast plastoglobules [18].

However, the signaling function of lipids has only been recently discovered and remains relatively poorly understood. Lipid signaling molecules participating in the plant response to stress include lysophospholipids (partially deacylated PL, products of phospholipase-catalyzed reaction), free fatty acids (FFAs), DAG, oxylipins (products of FA enzymatic or nonenzymatic oxidation), sphingolipids, phosphoinositides (phosphorylated PI derivatives), and PA [19].

## 3. A Brief Overview of the Plant Defense System

Plants have evolved complex passive and active defense mechanisms to protect themselves against pathogen infection. Passive resistance mainly relies on physical barriers on the cell surface and antimicrobial substances, termed phytoanticipins, present in plant tissues before infection [20,21,22,23].

To establish a pathogenic relationship with the host plants, pathogens must overcome the preformed structural barriers protecting the cells. The first barrier presented to the pathogen is the lipophilic cuticle deposited onto the outer side of the epidermal cell wall. This protective shield formed by the cuticle helps limit pathogen attachment, invasion, and infection. However, its role in plant–pathogen interactions goes beyond being a passive physical barrier for the microbes that attack plants [23,24].

The structural and chemical properties of the cuticle and the cell wall have a strong influence on plant–pathogen interactions. Studies on mutants with cuticle and cell wall structure alterations reveal a correlation between the altered cuticle and cell wall chemical composition and plant susceptibility to pathogens [25,26]. Plants have evolved abilities to monitor the integrity of cuticles and cell walls under biotic stress to activate innate immune responses [25,27]. The cuticle and cell wall form a continuum, an interface of plant–microbe interactions which undergoes dynamic remodeling in response to pathogen attack. Such changes generate danger signals which initiate defense responses [28]. These signals are immunogenic factors originating from the host plant or the invading pathogen; they are recognized by receptors and activate immune signaling pathways. Thus, pathogen-induced alterations in the cuticle–cell wall continuum are interconnected with the plant immune system. The first line of induced defense is activated following the recognition of pathogen-associated molecular patterns (PAMPs) or damage-associated molecular patterns (DAMPs) by the plasma membrane’s pattern recognition receptors (PRRs), leading to pattern-triggered immunity (PTI), providing basal immunity against entire classes of pathogens [29]. PAMPs are derived from the pathogen and are highly conserved molecules of diverse chemical structures serving as molecular fingerprints of pathogens. PRR-mediated immune responses can also be initiated by DAMPs, endogenous molecules released from the damaged plant tissue. DAMP-triggered immunity shares overlapped signaling components with PTI, and DAMPs can function as PTI amplifiers [30]. 

The second line of immune defense is triggered by the recognition of the avirulence (*Avr*) genes-coded pathogen effectors by plant resistance (*R*) gene products; this is known as effector-triggered immunity (ETI). ETI is highly species- and race-specific and more robust than PTI. It is activated upon recognition of pathogen effectors by intracellular nucleotide-binding domain leucine-rich repeat-containing receptors (NLRs) and involves the local hypersensitive response (HR), often followed by systemic resistance in the host plant [29]. 

One of the earliest manifestations of induced defense responses in plants is the HR, which occurs at the point of pathogen ingress. This specific type of programmed cell death (PCD) is generally associated with resistance to biotrophic pathogens, which grow and reproduce in living tissues. It may, however, benefit necrotrophs requiring dead host tissues to complete their life cycle [31]. 

The microbe recognition events taking place at the site of infection activate downstream signaling cascades. These produce mobile immune signals which are transported to systemic tissues, where they induce systemic resistance classified as systemic acquired resistance (SAR) and induced systemic resistance (ISR). Leaf pathogens induce SAR, whereas beneficial microbes interacting with roots induce ISR [32]. The systemic immune signals consist of compounds of different chemical structures, including proteins, lipid-derived molecules, and hormone-like compounds [33]. SAR is an induced immune mechanism conferring durable, broad-spectrum resistance with no specificity to the initial infection. It is characterized by elevated pathogenesis-related (*PR*) gene expression levels and the crosstalk between salicylic acid (SA)- and JA-dependent signaling pathways at the molecular level. SAR was also suggested to be inherited epigenetically, which has been referred to as “transgenerational SAR” [34,35].

Despite being activated by different mechanisms, PTI and ETI share some downstream effects. They include reactive oxygen species (ROS) generation, increased levels of intracellular Ca^2+^, synthesis of phytoalexins, activation of mitogen-activated protein kinases (MAPKs) cascades, transcriptional induction of defense genes, and hormone signaling with SA, JA, and ethylene, comprising the backbone of plant immunity [32,36]. The final resistance output, therefore, results from PTI-ETI crosstalk and increasing evidence shows that PTI and ETI should no longer be viewed as two separate branches of induced plant immunity [37,38].

## 4. Plant Surface Lipids in Plant–Pathogen Interactions

The leaf epicuticular wax and cutin are generally considered the first barriers to the invading pathogen in plants. They can also serve as a reservoir of signals that trigger defense responses in host plants or are utilized by pathogens to facilitate their infection process [39]. The chemical composition and structure of epicuticular waxes have been found to determine fungal pathogen development and pathogenicity [40,41]. The abundance of epicuticular wax crystals is positively correlated with the efficiency of the leaf self-cleaning mechanism known as the “lotus effect”, which also promotes the washing of fungal spores from the leaf surface before germination and reduces the probability of infection by fungal pathogens targeting the leaves [42]. The cuticle provides a hydrophobic barrier that reduces the diffusion of water and nutrients to the plant surface, thus hindering the movement of foliar bacterial pathogens toward the natural openings through which they enter the plant. However, some *Pseudomonas* strains have evolved to produce hygroscopic surfactants that increase the diffusion of water across the cuticle and the wetness of the leaf surface, effectively overcoming this barrier [43]. 

While many attributes of the cuticle have been linked to altered susceptibility and resistance to pathogens, the relationship between pathogen resistance and the amounts of epicuticular waxes and cutin remains elusive [44,45]. Removing the epicuticular wax layer in some plant–pathogen systems influenced the defense response against pathogens. For instance, after wax removal, the resistant cultivar of *Brassica napus* exhibited significantly more severe disease symptoms following *Sclerotinia sclerotiorum* infection. No significant difference in disease severity was observed in the susceptible cultivar, suggesting that epicuticular wax was correlated to defense against *S. sclerotiorum* in the resistant cultivar but not in the susceptible one [44]. 

The chemical characteristics of waxes were essential in varying the pathogenicity of *Culvularia eragrostidis* on *Digitaria sanguinalis* (host) and *Festuca arundinacea* (nonhost). The components of the nonhost plant wax successfully prevented the fungal spores from adhesion. They were only partially degraded by extracellular esterases secreted by the pathogen in the infection process. The opposite was true for the *C. eragrostidis*–*D. sanguinalis* interaction, where the host plant wax enhanced the growth of *C. eragrostidis* germ tubes, and the pathogen esterases completely degraded the plant epicuticular wax [46]. The wax crystallization pattern and its chemical composition influenced the germination rate of *Erysiphe pisi* on pea leaves, triggering the early steps of biotrophic fungal development in its host plant [47]. Loss of abaxial leaf epicuticular wax in *Medicago truncatula* irg1/palm1 mutants resulted in reduced preinfection structure differentiation of the anthracnose pathogen *Colletotrichum trifolii* and two nonhost rust pathogens: *Phakopsora pachyrhizi* and *Puccinia emaculata* [48]. Very-long-chain (VLC) aldehyde wax constituents, especially n-hexacosanal (C26-aldehyde), a minor component of barley leaf wax, stimulated the barley powdery mildew (*Blumeria graminis* f.sp. *hordei*) conidia germination and differentiation in a dose- and chain-length-dependent manner in vitro [40]. Accordingly, in the *glossy11* wax mutant of maize, devoid of the VLC aldehydes from the leaf cuticular wax, the germination and differentiation of barley powdery mildew conidia on the leaves were restricted. However, the prepenetration process was restored by spraying the leaves with n-hexacosanal [41]. Octacosanal, the C28-aldehyde of wheat, induced the formation of appressoria and infection hyphae of the rust fungus (*Puccinia graminis* f.sp. *tritici*) in vitro, confirming that the pathogens can sense the components of the plant surface and adapt their pathogenesis accordingly [49]. In addition to wax aldehydes, wax primary alcohols and VLC alkanes were also morphogenetically active and involved in plant–host recognition by the pathogen, spore germination, and infection structure differentiation [50,51,52]. 

Apart from their role in initiating the preinvasion and infection processes, waxes are also involved in activating plant innate immune responses, as their biosynthesis can be initiated at the gene level by the presence of pathogens. VLCFAs, the major components of plant epicuticular waxes, are synthesized by the endoplasmic-reticulum-localized fatty acid elongase (FAE) complex, which consists of four core enzymes: 3-ketoacyl-CoA synthase, 3-ketoacyl-CoA reductase, 3-hydroxyacyl-CoA dehydratase, and enoyl-CoA reductase. The FAE complex transfers two carbon units from malonyl-CoA to the 18-carbon-CoA, providing a 20-carbon chain which can be further elongated to yield wax precursors between 20 and 38 carbons in length [53]. Raffaele et al. [54] indicate that the enzymes forming the FAE complex are potential targets of MYB30, a transcription factor acting as a positive regulator of PCD during the HR response in *Arabidopsis*. The authors proposed that during the incompatible interactions between *Arabidopsis* and avirulent bacterial pathogens, MYB30 modulates HR via VLCFAs as the cell death messengers. Similarly, the apple MdMYB30 transcription factor modulated resistance against the fungal pathogen *Botryosphaeria dothidea* by regulating apple cuticular wax biosynthesis via the activation of 3-ketoacyl-CoA synthase-encoding gene expression [55].

The relationship between cuticle structure and function in plant immune responses was confirmed in interactions with cuticle-degrading pathogens. Various *Arabidopsis* and *Solanum lycopersicum* mutants display modifications in cuticle structure related to its increased permeability, accompanied by a constitutive production of ROS; this was found to correlate with enhanced resistance to *Botrytis cinerea*, the causative agent of grey mold [56]. In *Arabidopsis* infected with *B. cinerea*, the induction of resistance concomitant to ROS accumulation was observed only under conditions where the cuticle permeability was increased. It has been proposed that the changes in the diffusive properties of the cuticle induced by infection may allow the early perception of MAMPs/DAMPs generated at the plant–pathogen interface and pathogen effectors, thus allowing faster and more efficient activation of PTI and ETI, respectively [39,57]. This concept, however, is still under debate, as some permeable cuticle mutants showed the opposite response, namely, increased susceptibility to fungal pathogens [58,59].

Most fungal pathogens secrete cutinases, i.e., nonspecific esterases that hydrolyze the cutin polyester and release free cutin monomers to overcome the cuticle’s structural barrier [60]. Although for some plant–pathogen interactions, the role of cutinases in fungal pathogenicity is still controversial, many studies on cutinase-deficient mutants confirmed their importance in pathogen virulence [61,62,63]. The genomes of individual species of fungal pathogens can possess genes encoding several cutinases, which are structurally and functionally diverse and expressed at different time points of pathogenesis [60,64,65]. In *Rhizoctonia cerealis*, the necrotrophic fungus causing sharp eyespot disease in several crops, one of the six cutinases, designated as RcCUT1, acts as an effector triggering necrosis, H_2_O_2_ accumulation and the expression of defense-related genes. Unlike other *R. cerealis* cutinases, RcCUT1 is expressed in all fungal infection and colonization stages and strongly contributes to fungal virulence [63]. Similarly, cutinase VdCUT11 of *Verticillium dahliae*, a highly virulent wilt fungal pathogen, was also shown to have activity as a necrotrophic effector required for pathogenicity [66]. In other studies, however, cutinases have not been conclusively implicated in the pathogenicity and virulence of fungal pathogens [67,68].

In the early stages of infection, the constitutively expressed cutinases secreted by the fungal spores alter the surface of the host plant. These promote successful adhesion and release small amounts of cutin monomers, which play diverse signaling roles in the interactions between host plants and pathogenic fungi. First, they transcriptionally induce the expression of fungal inducible cutinase genes, producing high enzyme levels [69]. The cutin monomers also trigger the formation of specialized fungal infection structures and cuticle penetration; the FAs liberated by plant cutin degradation can be used by the fungi as a carbon and energy source [70]. These processes facilitate the penetration of the fungus through the cuticle and favor infection progression [23,63]. 

Studies on *Arabidopsis* cuticular mutants infected with *Pseudomonas syringae* showed that cuticle-derived signals, such as cutin monomers or FAs related to cutin monomers, can change bacterial gene expression and modify the plant–pathogenic bacteria interaction [71]. Moreover, the cuticle plays an active role in SAR-related signaling. For example, an acyl carrier protein *ACP4*, required for cuticular C16 or C18 FA biosynthesis in leaves, is indispensable for perceiving the mobile SAR signal in distal tissues of *Arabidopsis*. The *ACP4* mutants were shown to generate a mobile SAR signal in lower leaves inoculated with bacteria but not to perceive it in the upper leaf. A mutation in *ACP4* compromises SAR due to the impairment of cuticle formation in the leaf [58].

Cutin monomers may also play a role in inducing immune responses in plants. Cutin monomers generated by the action of pathogen cutinases and cutinolytic lipases during the infection process serve as DAMPs. As immunogenic signals, they activate plant defenses against pathogens named PTI [39]. For example, the application of cutin monomers induced an array of plant defense responses, including ROS generation and PR gene expression, enhancing resistance to *B. cinerea*, *E. graminis*, *E. Graminis* f.sp. *hordei*, and *Magnaporthe grisea* [72,73,74]. However, the recognition process and receptors for cutin monomers remain unknown [75].

## 5. Free Fatty Acids and Phytooxylipins in Plant–Pathogen Interactions

During biotic stress, membrane lipids undergo significant modifications, and lipases are among the most stress-responsive enzymes that modify membrane lipids [1]. They liberate FFAs, such as C18:0, C18:1, C18:2, and C18:3, which contribute to defense against pathogens acting either directly on the microbes or indirectly as oxylipins [76]. 

FFAs exhibit antimicrobial activity by penetrating and disrupting biological membranes. They mainly target bacterial cell membranes, increasing membrane permeability, disrupting the electron transport chain, interfering with oxidative phosphorylation, and inhibiting membrane-associated enzymes and nutrient uptake. This leads to cellular content leakage and bacterial cell death, compromising the spread of the pathogens in infected tissue [77]. FFAs with medium (8–12 carbon atoms) to longer (>12 carbon atoms) chains are particularly effective against Gram-positive bacteria, whereas those containing six carbons or fewer affect Gram-negative bacteria. For long-chain unsaturated FFAs, the antimicrobial activity increases with the chain length and degree of unsaturation [78].

FFAs also act as antifungal agents. Saturated FA exerted antifungal activity towards devastating phytopathogenic fungi, namely, *Alternaria solani*, *Colletotrichum lagenarium*, and *Fusarium oxysporum*, by inhibiting spore germination and mycelium growth [79]. Unsaturated FAs, namely, oleic acid (C18:1), C18:2, and C18:3, reduced the mycelial growth of *R. solani*, *Pythium ultimum*, and *Pyrenophora avenae* in vitro [80]. It is believed that their antifungal activity acts though inducing membrane disorder and cytoplasmic disintegration [81]. 

Trienoic acids (TAs), especially C18:3 derived from chloroplasts, act as effective activators of NADPH oxidase. In *Arabidopsis*, ω-3 fatty acid desaturases FAD7 and FAD8 double mutants (*fad7fad8*), lacking plastidial ω-3 desaturase activity, reduced the accumulation of TAs observed in the chloroplast membranes. Consequently, the NADPH-mediated ROS generation and their signaling role in the early stages of plant–pathogen interaction are reduced. These mutants show decreased defense against several avirulent *P. syringae* strains, suggesting that the defense response depends on chloroplast C18:3, which may play an important role in regulating the strength of HR [82,83].

The Δ-9 stearoyl acyl carrier protein desaturase (SAD) catalyzes the conversion of stearic acid (C18:0) to C18:1 and is crucial in regulating cellular PUFA content [83]. Avocado fruits with increased SAD are more resistant to *C. gloeosporioides*. In contrast, the SAD-defective *Arabidopsis* suppressor of SA insensitivity (*ssi*) mutants, with high 18:0 and reduced total 18:1 levels, show constitutive expression of PR genes, SA accumulation, susceptibility to the necrotroph *B. cinerea*, and enhanced resistance to the biotrophs *P. syringae* and *P. parasitica*. Based on these and other studies, 18:1 FA levels were suggested to modulate defense responses in *Arabidopsis* [84,85,86]. 

**Figure 2:**
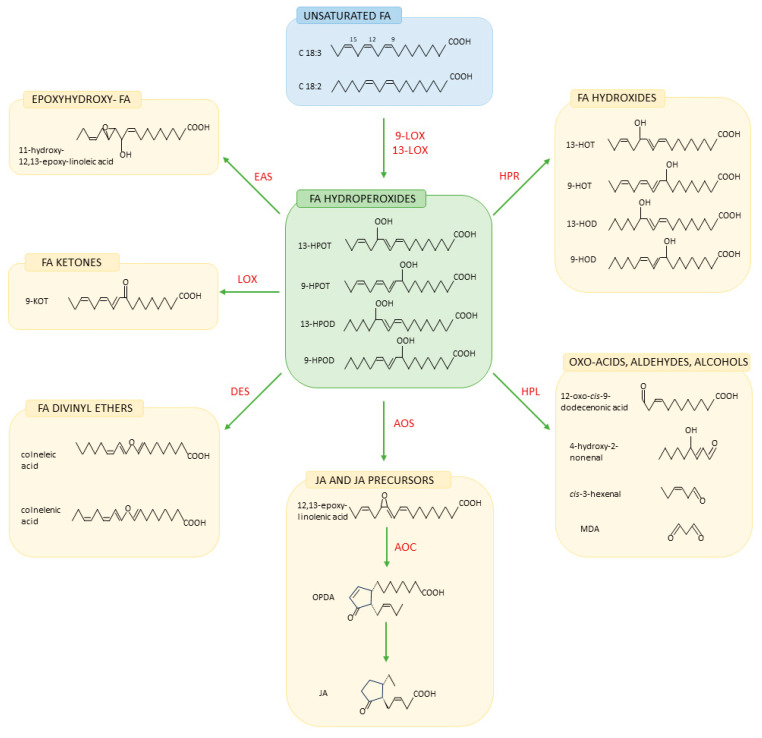
Oxylipin biosynthesis. Abbreviations: LOX: lipoxygenase; HPR: hydroperoxide reductase; HPL: hydroperoxide lyase; AOS: allene oxide cyclase; AOC: allene oxide cyclase; DES: divinyl ether synthase; EAS: epoxy alcohol synthase; FA: fatty acid; 13-HPOT: 13-hydroperoxy octadecatrienoic acid; 9-HPOT: 9-hydroperoxy octadecatrienoic acid; 13-HPOD: 13-hydroperoxy octadecadienoic acid; 9-HPOD: 9-hydroperoxy octadecadienoic acid; 13-HOT: 13-hydroxy octadecatrienoic acid; 9-HOT: 9-hydroxy octadecatrienoic acid; 13-HOD: 13-hydroxy octadecadienoic acid; 9-HOD: 9-hydroxy octadecadienoic acid; MDA: malondialdehyde; OPDA: 12-oxo-phytodienoic acid; JA: jasmonic acid; 9-KOT: 9-keto-octadecatrienoic acid.

Phytooxylipins are plant bioactive lipids formed enzymatically or via nonenzymatic oxygen-dependent oxidation pathways from PUFAs such as C18:2 and C18:3. In 16:3 plants, including *Arabidopsis*, C16:3 is also an important substrate for phytooxylipin synthesis [87]. In most cases, the enzymatic oxidation of PUFAs occurs in reactions catalyzed by lipoxygenases (LOXs) generating FA hydroperoxides. LOXs are categorized as 9-LOX, 13-LOX, and 9/13-LOX (mixed regiospecificity), depending on their ability to incorporate oxygen at the 9- or 13-hydrocarbon positions in the FA chain. C18:2 and C18:3 are oxygenated to produce C18:2 hydroperoxides, e.g., 9-hydroperoxy octadecadienoic acid (9-HPOD) and 13-hydroperoxy octadecadienoic acid (13-HPOD), or C18:3 hydroperoxides such as 9-hydroperoxy octadecatrienoic acid (9-HPOT) and 13-hydroperoxy octadecatrienoic acid (13-HPOT) (Figure 2). 

LOX-derived hydroperoxides may be converted by various enzymes, giving rise to numerous oxylipins. Allene oxide synthase (AOS) catalyzes the dehydration of FA hydroperoxides to form unstable allylic epoxides (allene oxides), which, in the presence of allene oxide cyclase (AOC), may be converted into 12-oxo-phytodienoic acid (OPDA) and subsequently to JA. Hydroperoxide lyase (HPL) catalyzes the cleavage of FA hydroperoxides to volatile aldehydes and oxo-acids. Depending on the substrate specificity of HPL, 6-carbon or 9-carbon aldehydes are produced from 13-hydroperoxides or 9-hydroperoxides, respectively. Subsequently, aldehydes spontaneously or enzymatically can be isomerized or converted to alcohols and hydroxy- or acetate-containing derivatives. FA hydroperoxides can also be substrates for LOX, hydroperoxide reductase (HPR), and epoxy alcohol synthase (EOS) and give rise to keto-, hydroxy-, and epoxyhydroxy derivatives, respectively [3,88,89].

JA, its precursor cyclized OPDA, and their derivatives, known as jasmonates, are the best-characterized LOX-derived metabolites studied in plants. Jasmonates play essential roles in regulating plant responses to environmental stimuli, including pathogens and insects [90]. JA is a key hormone player in the plant defense network, and its interaction with SA shapes the plant immune response [91,92]. Jasmonates are generally involved in plant resistance against necrotrophic pathogens. JA and its isoleucine conjugate (JA-Ile) trigger massive reprogramming of the gene expression required to activate defense responses. Consequently, plants defective in JA synthesis or JA reception show altered responses to pathogens [93]. The importance of JA and JA-mediated signaling in plant–pathogen interactions has been covered in several recent reviews, and therefore will not be discussed here [90,93,94,95,96]. 

Apart from the role of JA in the defense signaling network, its precursor OPDA has been reported to trigger autonomous responses which do not depend on its conversion to the hormone JA [97]. It has been confirmed that OPDA plays a regulatory role independent of JA. For example, in transgenic tomato plants accumulating less OPDA, infection with the fungus *B. cinerea* resulted in lowered resistance due to decreased callose accumulation at the infection sites [98]. In rice, however, OPDA functioned as a signal for producing phytoalexins [99]. OPDA, synthesized from C18:3 in chloroplasts, was found to trigger retrograde signaling towards the nucleus, which stimulates and coordinates defense gene expression [100]. In *B. cinerea*-infected tomato plants, this increased the local defense capacity, manifested by OPDA-mediated stimulation of callose deposition at the infection sites, preventing the cell-to-cell spread of the pathogen. In this interaction, OPDA was also required to activate basal defense in tomato plants fully [98]. 

In addition to its role in local defense, it has been proposed that OPDA may be involved in long-distance signaling, allowing the systemic propagation of immunity. OPDA and 9,10-ketol-octadecadienoic acid (KODA) are two xylem-mobile oxylipins found to be associated with *Trichoderma virens*-induced resistance (ISR). In maize, the accumulation of OPDA and KODA, rather than JA, was indispensable for ISR against *C. graminicola* [101].

Our knowledge of how OPDA is perceived for signaling and its mode of action, e.g., the interaction with the redox network, remains elusive [102,103]. It was found that the expression profiles of genes involved in stress responses, detoxification, and secondary metabolism induced by JA and OPDA were different [104]. Moreover, in *Arabidopsis*, OPDA was found to occur not only as a free acid but also as esters with galactolipids, such as MGDG and DGDG, known as arabidopsides [97,105]. These OPDA-containing galactolipids were shown to have multifaceted functions in plant–pathogen interactions. They are synthesized during the HR response triggered by bacterial effectors, act directly as antimicrobial substances inhibiting pathogen growth, and function as storage compounds allowing the slow release of free OPDA, which is then converted to JA [100]. In addition to the OPDA-containing galactolipids, OPDA-containing SQDG, PI, and PG species have also been reported to accumulate during the HR response in *Arabidopsis* [106].

In plants, OPDA signaling is also involved in regulatory mechanisms that mediate switching between growth and defense under biotic stress; this is an important consideration because investing available resources in defense mechanisms rather than plant growth will increase plant resistance [107]. The molecular model of this growth-defense trade-off relies on cyclophilin 20-3 (CYP20-3), a plastidial OPDA-binding protein. In chloroplasts, CYP20-3 is a crucial regulator at the interface between the OPDA-dependent redox-mediated retrograde signaling (defense) and light-dependent reduction in toxic by-products in photosynthesis and activation of Calvin cycle enzymes (growth). This mechanism has been proposed to fine-tune the allocation of resources between growth and defense responses [107,108].

13-LOX-produced phytooxylipins include C6 green leaf volatiles (GLVs) and C5 pentyl leaf volatiles (PLVs) [109]. Unlike the GLVs, which are known for their signaling role in plant-to-plant and plant-to-insect communication, the biosynthesis and function of PLVs in plants remain obscure [109,110]. Although GLVs and PLVs are often coemitted, they induce antagonistic biological effects. In maize, PLVs were found to induce resistance to anthracnose disease caused by *C. graminicola* by activating the production of oxylipin ketols, whereas GLVs promoted this disease progression through induction of JA and suppression of SA [109,111]. PLVs are suggested to activate resistance to biotrophic and hemibiotrophic pathogens. For example, 3-pentanol, representing PLV, triggered resistance to *P. syringae* pv *lachrymans* in cucumber [112]. In *Arabidopsis*, this volatile compound primed plant systemic resistance against *P. syringae* pv *tomato* via SA- and JA-dependent signaling [113]. Contrasting results were obtained in tomato where both C5 and C6 volatile compounds were not important in defense against bacterial pathogen *Xanthomonas campestris* pv *vesicatoria* [114].

In addition to the importance of 13-LOX-generated oxylipins in plant defense against pathogens, the participation of the 9-LOX oxylipin pathway, likely by modulating the hormonal response, was also revealed. For example, 9-LOX-produced 9-ketooctadecatrienoic acid (9-KOT) oxylipin accumulated in *Arabidopsis* leaves responding to avirulent *P. syringae* pv *tomato* DC3000 and contributed to controlling bacterial infection. It has been proposed that 9-KOT interferes with hormonal changes induced by bacterial effectors; indeed, about 50% of the genes upregulated after 9-KOT treatment were found to corresponded with genes responding to *Pseudomonas* infection, indicating a protective effect of this oxylipin [115]. In cultured potato cells, treatment with *Phytophthora infestans* elicitor preferentially stimulated the 9-LOX pathway, which led to the accumulation of divinyl ether colneleic acid, an antimicrobial oxylipin formed from 9-hydroperoxide. In this system, neither 13-LOX activity nor 13-LOX products were induced in response to elicitation [116]. 

The importance of 9-LOX for resistance was confirmed in transgenic plants. Overexpression of *LOX1*, a 9-LOX gene, in tobacco was sufficient to reduce the host plant’s susceptibility to virulent races of *P. parasitica* var *nicotianae* [117]. However, Fauconnier et al. [118] found no correlation between oxylipin synthesis rates and concentrations and resistance to *P. infestans*, despite the activation of the 9-LOX pathway and oxylipin accumulation in potato leaves infected with *P. infestans*. These results suggest that specific oxylipins could have different effects on different plant species, and their mechanism of action requires further study.

It has also been proposed that the 9-LOX pathway is essential for forming azelaic acid, a nine-carbon dicarboxylic acid generated by oxidative cleavage of C18 unsaturated fatty acids with a double bond at C9, which was suggested to be a general oxidative stress signal implicated in SAR [119]. Azelaic acid precursors are derived from the MGDG and DGDG galactolipids of the chloroplast membrane. DGDG is required for the pathogen-induced accumulation of SA and nitric oxide (NO), which act as signaling molecules during SAR. MGDG regulates the biosynthesis of azelaic acid and glycerol-3-phosphate, which function downstream of NO. DGDG, synthesized in the outer membrane of plastids, is also involved in retrograde signaling between the chloroplast and the nucleus by inducing the expression of nuclear genes, including *SLD2* and *EDS5* genes involved in SA biosynthesis and transport, respectively [120,121].

In addition to the enzymatically-formed oxylipins, others are produced nonenzymatically from C18:3 via the action of ROS, such as phytoprostanes; these also accumulate in pathogen-challenged plants as a consequence of oxidative stress and serve as active signaling molecules. They are believed to contribute to ROS signal transduction as intracellular second messengers during plant–pathogen interactions, and to induce the production of phytoalexins, antimicrobial secondary metabolites. For example, in peroxide-stressed tobacco cell cultures and tomato plants infected with *B. cinerea*, phytoprostanes induced MAPK and phytoalexin synthesis, thus enhancing biotic stress tolerance [122]. Phytoprostanes have been shown to induce defense genes involved in secondary metabolism and detoxification, such as those encoding glutathione-S-transferase (GST) [123].

Oxylipins regulate many aspects of bacterial and fungal lifestyles, quorum sensing, and interactions with the host plant, including manipulating plant defense reactions for enhancing host resistance. Plant oxylipins interfere with pathogen growth and reproduction, signal pathogen attacks, and act as antimicrobial agents [124]. For example, plant oxylipins from peanut seeds alter sporulation and mycotoxin synthesis in invading *Aspergillus*; however, oxylipins endogenously produced by *Aspergillus*, which are involved in sporulation and mycotoxin production, also inhibit peanut seed LOX gene expression, possibly altering the host plant–fungus interaction [125]. Thus, oxylipins mediate a reciprocal cross-talk in the *Aspergillus*-seed pathosystem [126]. As fungal, bacterial, and plant oxylipins share some structural and functional similarities, the host plant and the pathogen can use the same oxylipins simultaneously. Therefore, during the plant–pathogen interaction, oxylipins act as inter-organismal signals involved in determining the outcome of the interaction [95,124,127,128]. 

Although some pathosystems demonstrate no correlation between plant resistance and phytooxylipin biosynthesis rates or concentrations [118], oxylipins are at the front line of plant–pathogen interactions due to their signaling role in both local and systemic defense: these are reviewed in [88,95,129]. In this context, the interplay of oxylipins with the plant redox network seems to be an essential element coupling pathogen-induced rapid synthesis of ROS and defense signaling. Phytooxylipins interact with the plant cell redox network, by inter alia modulating the amounts and activities of antioxidant enzymes concerned with ROS synthesis (superoxide dismutase) and scavenging (catalase). They also interact with redox sensors, redox transmitters, such as thioredoxins and glutaredoxins, and redox target proteins, e.g., GST and CYP20-3 [102]. 

Phytooxylipins also provide local defense against pathogens. In *Arabidopsis*, recognition of *P. syringae* avirulence protein activated the 13- and 9-LOX-dependent oxylipin biosynthesis pathways during HR-associated cell death [130]. It has been suggested that rather than simply being the products of oxidative plant cell death, HR-associated phytooxylipins may mediate plant defense [88,115,120]. 

Phytooxylipins also trigger gene expression, and phytooxylipin treatment has been found to demonstrate a similar transcriptional response to that induced by avirulent bacteria. One of the immune response genes induced was the PR gene *HEL* (hevein-like protein, PR4). Interestingly, the nonenzymatically produced volatile oxylipin, acrolein, was a much more active *HEL* gene stimulator than larger alkenal homologs, like 2-hexenal [130].

## 6. Other Lipids and Lipid-Related Mediators Involved in Plant–Pathogen Interactions

Plant lipid transfer proteins (LTPs), members of the PR protein family (PR14), appear to function as regulators of immune processes in many plant–pathogen interactions [131]. Their importance in the immune system is illustrated by the fact that LTP expression is inducible by infection with pathogens, and transgenic *Arabidopsis* plants overexpressing an *LTP* gene showed increased resistance to *A. alternata* and *B. cinerea* [132]. Transgenic rice plants expressing a nonspecific *LTP* gene secreted to the apoplast space exhibited enhanced resistance to *M. grisea*, *R. solani*, and *X. oryzae* [133]. 

LTPs can inhibit the growth of bacterial and fungal pathogens, and this antimicrobial effect may result from the permeabilization of cell membranes. They are also involved in the response and signaling of the stress hormones SA, JA, and abscisic acid (ABA). For example, in *Arabidopsis*, LTP3 negatively modulated plant resistance to *P. syringae* pv *tomato* by manipulating the ABA-SA hormonal balance [134]. LTPs also interact with JA, and the resulting LTP-JA complexes bind to plasma-membrane-located elicitin receptors to activate immune responses. Exogenous application of LTP-JA promotes resistance to *B. cinerea* and *P. infestans* [135,136]. LTPs also contribute to pathogen resistance by regulating epicuticular wax accumulation and maintaining adhesion integrity between the cuticle and the plant cell wall [137]. Finally, they are involved in transferring long-distance SAR signals via the phloem. SAR establishment in *Arabidopsis* requires long-distance translocation of LTPs, namely, AZI1 (Azelaic Acid Induced 1) and DIR1 (Defective in Induced Resistance 1) in conjunction with glycerol-3-phosphate [137]. Loss of AZI1 function resulted in loss of systemic immunity triggered by the pathogen or azelaic acid [138]. Moreover, the nonspecific LTP (PR14) interacts with PR1 in the apoplastic space to increase the antimicrobial activity of PR1 in an ROS-dependent manner and, thus, pathogen resistance [131]. 

Whereas oxylipins are the best-known signaling lipids, other lipids, such as sphingolipids, PA, and DAG, have also been proposed to mediate biotic stress defenses in plants. 

Cers, a class of sphingolipids, are involved in the defense against bacterial and fungal pathogens by inducing PCD in plants. The Cer core consists of two structural moieties: the sphingoid LCB and the FA chain linked via an amide bond. Mutations resulting in increased Cer contents caused enhanced SA-dependent plant cell death during ETI [139]. The ETI in plants involves the accumulation of LCBs as an early signaling step upstream of SA. LCBs activate MPK6, an MAPK from the ETI signaling pathway, which promotes SA-dependent cell death, namely, HR as a defense against (hemi)biotrophic pathogens. Interestingly, mycotoxins secreted by necrotrophic fungi *A. alternata* and *Fusarium* species inhibit acyl-CoA-dependent Cer synthase, leading to Cer depletion and inducing host cell death for the benefit of the pathogen. This mode of mycotoxin action was suggested as a virulence strategy of necrotrophic pathogens. Thus, the connection between Cers and PCD in plants is associated with plant disease caused by necrotrophs and defense against biotrophs, indicating that Cers play a positive and a negative role in plant immunity, depending on the type of pathogen [140]. 

PA, an intermediate in glycerolipid synthesis, accumulates in response to many stresses, including plant–pathogen interactions [19]. Its accumulation and signaling can be triggered by phospholipase D (PLD)-mediated hydrolysis of structural phospholipids like PC or via phospholipase C (PLC), which generates DAG that is phosphorylated to PA by DAG kinase (DGK). PA binds and stabilizes the NADPH oxidase RESPIRATORY BURST OXIDASE HOMOLOG D, regulating ROS production in plant PTI and ETI. In *Arabidopsis*, the cellular homeostasis of PA intertwined with ROS production is suggested to be regulated by phosphorylation of DGK5 [141]. PA released by the PLC-DGK pathway is an early signal in pathogen-elicitor-induced defense responses. In tobacco cell suspensions, the race-specific protein elicitor AVR4 from *Cladosporium fulvum* triggered PA-mediated signaling, leading to defense responses such as oxidative burst. The signaling role of PA was supported by its rapid conversion to diacylglycerol pyrophosphate, which only occurs under conditions that activate signaling [142]. PLD activity and gene expression were induced in plants infected by bacterial and fungal pathogens, and PLD was found to play both positive and negative roles in plant immunity depending on the lifestyle of the pathogen, as well as the specific PLD involved and its interaction with stress hormones; a suitable review is provided by Li and Wang [143].

Another aspect of phospholipid involvement in the plant immune response is related to defense protein sorting in plants. After a pathogen is perceived by membrane receptors, an immune response is activated through the protein/membrane trafficking network, which enables an effective transport of defense-associated molecules to subcellular compartments [144]. Phospholipids and related enzymes have been found to play a crucial role in these processes. Coordination of phospholipid-based signaling and membrane trafficking, especially at the plasma membrane, is essential for pathogen recognition and activation of the immune system. For example, phosphatidyl-inositol-3-phosphate [PtIns(3)P] vesicle transport is necessary for the secretion of defense PR1 proteins to the apoplastic space where they exhibit their antimicrobial activity [145]. Upon pathogen recognition, PRRs bind to bacterial or fungal PAMP, and the ligand-receptor complex is internalized from the plasma membrane by endocytosis. This internalization is facilitated by PA and phosphatidylinositol-4,5-bisphosphate [PtIns(4,5)P2] targeting the PRR-PAMP complex [144]. In *Arabidopsis*, PLD and PLD-generated PA was found to accumulate in papillae, i.e., at the pathogen entry sites, after infection by the biotrophic fungus *B. graminis* f.sp. *hordei*. The PA in the papillae recruited effector proteins such as NADPH oxidase, thus initiating PA-related defense signaling and preventing penetration by the pathogen [146,147]. The examples above support the importance of phospholipid-based signaling and its coordination with defense protein sorting to efficiently activate immune responses in plants.

The complex interplay among various phospholipases, DGKs, stress hormones, and defense-related molecules reveals the emerging role of PA as an important secondary messenger in plant defense mechanisms against microbial pathogens [4,76].

## 7. Concluding Remarks and Future Perspectives

**Figure 3:**
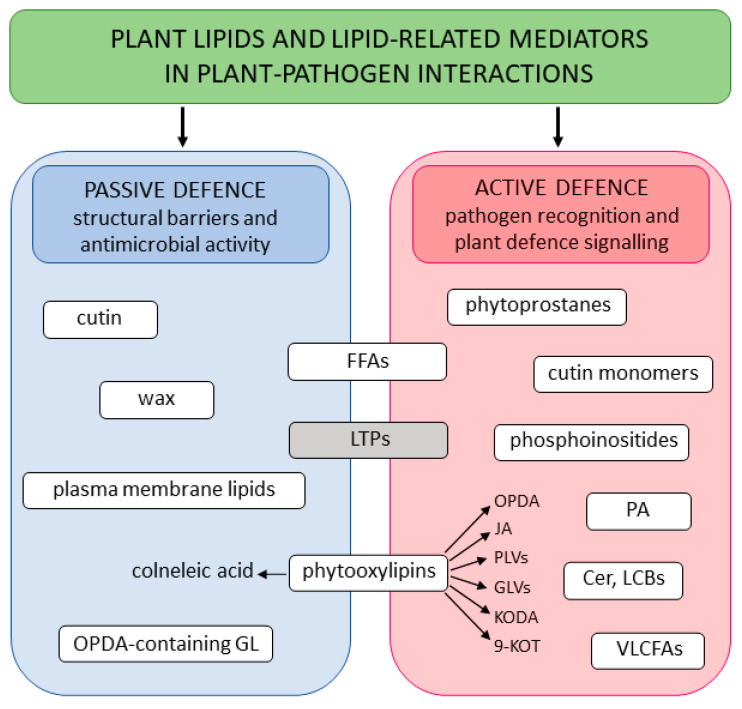
The function of plant lipids and lipid-related mediators in plant–pathogen interactions described in the present work. Abbreviations: OPDA: 12-oxo-phytodienoic acid: GL: galactolipid; FFA: free fatty acid; LTPs: lipid transfer proteins; JA: jasmonic acid; KODA: 9,10-ketol-octadecadienoic acid; 9-KOT: 9-keto-octadecatrienoic acid; Cer: ceramide; LCBs: long chain bases; PA: phosphatidic acid; VLCFAs: very-long-chain fatty acids; GLVs: green leaf volatiles; PLVs: pentyl leaf volatiles.

In plants, lipids are involved in plant development and the stress response, and play many essential structural, storage, signaling, and defensive roles. Under biotic stress, lipids enhance the passive defense against pathogens by acting as structural elements of the penetration barriers and antimicrobials. They also play critical roles in activating a range of pathogen-induced immune responses, and contribute to pathogen recognition, local and systemic stress signal transmission, and defense gene induction (Figure 3). Lipid signals are also indispensable for facilitating plant–microbe communication. 

Although our understanding of the complex role of lipids and lipid-derived signals in plant–pathogen interactions is still expanding, many gaps still exist. These include the molecular mechanisms of lipid-based signaling and its importance for plant resistance to pathogens. Elucidating the specific functions of different lipids and lipid-derived signals in the matrix of the plant immune system remains a future challenge. Considering the versatile role of lipids in plant immunity, this would provide powerful theoretical support for improving disease resistance in crops.

Future climate change is likely to impact pathogen spread, disease incidence, and severity, increasing the risk to global food security. Therefore, there is an urgent need to develop climate-resilient crop plants with enhanced resistance to pathogens. In this context, deciphering the role of lipids and lipid derivatives in plant immune mechanisms can aid strategies designed for the simultaneous improvement of disease resistance and plant performance.

## Data Availability

Not applicable.

## References

[1] Shah J. (2005). Lipids, Lipases, and Lipid-Modifying Enzymes in Plant Disease Resistance. Annu. Rev. Phytopathol..

[2] Siebers M., Brands M., Wewer V., Duan Y., Hölzl G., Dörmann P. (2016). Lipids in Plant–Microbe Interactions. Biochim. Biophys. Acta Mol. Cell Biol. Lipids.

[3] Pretorius C.J., Zeiss D.R., Dubery I.A. (2021). The Presence of Oxygenated Lipids in Plant Defense in Response to Biotic Stress: A Metabolomics Appraisal. Plant Signal. Behav..

[4] Seth T., Asija S., Umar S., Gupta R. (2024). The Intricate Role of Lipids in Orchestrating Plant Defense Responses. Plant Sci..

[5] Cavaco A.R., Matos A.R., Figueiredo A. (2021). Speaking the Language of Lipids: The Cross-Talk between Plants and Pathogens in Defence and Disease. Cell. Mol. Life Sci..

[6] Reszczyńska E., Hanaka A. (2020). Lipids Composition in Plant Membranes. Cell Biochem. Biophys..

[7] Kehelpannala C., Rupasinghe T., Hennessy T., Bradley D., Ebert B., Roessner U. (2021). The State of the Art in Plant Lipidomics. Mol. Omics.

[8] Shimojima M. (2011). Biosynthesis and Functions of the Plant Sulfolipid. Prog. Lipid Res..

[9] Hölzl G., Dörmann P. (2019). Chloroplast Lipids and Their Biosynthesis. Annu. Rev. Plant Biol..

[10] Michaelson L.V., Napier J.A., Molino D., Faure J. (2016). Plant Sphingolipids: Their Importance in Cellular Organization and Adaption. Biochim. Biophys. Acta.

[11] Mamode Cassim A., Gouguet P., Gronnier J., Laurent N., Germain V., Grison M., Boutté Y., Gerbeau-Pissot P., Simon-Plas F., Mongrand S. (2019). Plant Lipids: Key Players of Plasma Membrane Organization and Function. Prog. Lipid Res..

[12] Furt F., Simon-Plas F., Mongrand S., Murphy A., Schulz B., Peer W. (2011). Lipids of the Plant Plasma Membrane. The Plant Plasma Membrane. Plant Cell Monographs.

[13] Macabuhay A., Arsova B., Walker R., Johnson A., Watt M., Roessner U. (2022). Modulators or Facilitators? Roles of Lipids in Plant Root–Microbe Interactions. Trends Plant Sci..

[14] Meï C., Michaud M., Cussac M., Albrieux C., Gros V., Maréchal E., Block M.A., Jouhet J., Rébeillé F. (2015). Levels of Polyunsaturated Fatty Acids Correlate with Growth Rate in Plant Cell Cultures. Sci. Rep..

[15] Popko J., Wenk M.R. (2017). Encyclopedia of Lipidomics. Encyclopedia of Lipidomics.

[16] Moreau P., Bessoule J.J., Mongrand S., Testet E., Vincent P., Cassagne C. (1998). Lipid Trafficking in Plant Cells. Prog. Lipid Res..

[17] Kunst L., Samuels L. (2009). Plant Cuticles Shine: Advances in Wax Biosynthesis and Export. Curr. Opin. Plant Biol..

[18] Lu J., Xu Y., Wang J., Singer S.D., Chen G. (2020). The Role of Triacylglycerol in Plant Stress Response. Plants.

[19] Okazaki Y., Saito K. (2014). Roles of Lipids as Signaling Molecules and Mitigators during Stress Response in Plants. Plant J..

[20] Anjali, Kumar S., Korra T., Thakur R., Arytselvan R., Kashyap A.S., Nehela Y., Chaplygin V., Minkina T., Keswani C. (2023). Role of Plant Secondary Metabolites in Defence and Transcriptional Regulation in Response to Biotic Stress. Plant Stress.

[21] Cook D.E., Mesarich C.H., Thomma B.P.H.J. (2015). Understanding Plant Immunity as a Surveillance System to Detect Invasion. Annu. Rev. Phytopathol..

[22] Bentham A.R., de la Concepcion J.C., Mukhi N., Zdrzałek R., Draeger M., Gorenkin D., Hughes R.K., Banfield M.J. (2020). A Molecular Roadmap to the Plant Immune System. J. Biol. Chem..

[23] Arya G.C., Sarkar S., Manasherova E., Aharoni A., Cohen H. (2021). The Plant Cuticle: An Ancient Guardian Barrier Set Against Long-Standing Rivals. Front. Plant Sci..

[24] Malinovsky F.G., Fangel J.U., Willats W.G.T. (2014). The Role of the Cell Wall in Plant Immunity. Front. Plant Sci..

[25] Bacete L., Mélida H., Miedes E., Molina A. (2018). Plant Cell Wall-Mediated Immunity: Cell Wall Changes Trigger Disease Resistance Responses. Plant J..

[26] Sela D., Buxdorf K., Shi J.X., Feldmesser E., Schreiber L., Aharoni A., Levy M. (2013). Overexpression of AtSHN1/WIN1 Provokes Unique Defense Responses. PLoS ONE.

[27] Wolf S. (2022). Cell Wall Signaling in Plant Development and Defense. Annu. Rev. Plant Biol..

[28] Gust A.A., Pruitt R., Nürnberger T. (2017). Sensing Danger: Key to Activating Plant Immunity. Trends Plant Sci..

[29] Zhou J.M., Zhang Y. (2020). Plant Immunity: Danger Perception and Signaling. Cell.

[30] Hou S., Liu Z., Shen H., Wu D. (2019). Damage-Associated Molecular Pattern-Triggered Immunity in Plants. Front. Plant Sci..

[31] Balint-Kurti P. (2019). The Plant Hypersensitive Response: Concepts, Control and Consequences. Mol. Plant Pathol..

[32] Vlot A.C., Sales J.H., Lenk M., Bauer K., Brambilla A., Sommer A., Chen Y., Wenig M., Nayem S. (2020). Systemic Propagation of Immunity in Plants. New Phytol..

[33] Ádám A.L., Nagy Z., Kátay G., Mergenthaler E., Viczián O. (2018). Signals of Systemic Immunity in Plants: Progress and Open Questions. Int. J. Mol. Sci..

[34] Luna E., Bruce T.J.A., Roberts M.R., Flors V., Ton J. (2012). Next-Generation Systemic Acquired Resistance. Plant Physiol..

[35] López Sánchez A., Pascual-Pardo D., Furci L., Roberts M.R., Ton J. (2021). Costs and Benefits of Transgenerational Induced Resistance in Arabidopsis. Front. Plant Sci..

[36] Klessig D.F., Choi H.W., Dempsey D.A. (2018). Systemic Acquired Resistance and Salicylic Acid: Past, Present, and Future. Mol. Plant-Microbe Interact..

[37] Yuan M., Ngou B.P.M., Ding P., Xin X.F. (2021). PTI-ETI Crosstalk: An Integrative View of Plant Immunity. Curr. Opin. Plant Biol..

[38] Naveed Z.A., Wei X., Chen J., Mubeen H., Ali G.S. (2020). The PTI to ETI Continuum in Phytophthora-Plant Interactions. Front. Plant Sci..

[39] Ziv C., Zhao Z., Gao Y.G., Xia Y. (2018). Multifunctional Roles of Plant Cuticle during Plant-Pathogen Interactions. Front. Plant Sci..

[40] Hansjakob A., Bischof S., Bringmann G., Riederer M., Hildebrandt U. (2010). Very-Long-Chain Aldehydes Promote in Vitro Prepenetration Processes of Blumeria Graminis in a Dose- and Chain Length-Dependent Manner. New Phytol..

[41] Hansjakob A., Riederer M., Hildebrandt U. (2011). Wax Matters: Absence of Very-Long-Chain Aldehydes from the Leaf Cuticular Wax of the Glossy11 Mutant of Maize Compromises the Prepenetration Processes of Blumeria Graminis. Plant Pathol..

[42] Beattie G.A., Marcell L.M. (2002). Effect of Alterations in Cuticular Wax Biosynthesis on the Physicochemical Properties and Topography of Maize Leaf Surfaces. Plant Cell Environ. Environ..

[43] Burch A.Y., Zeisler V., Yokota K., Schreiber L., Lindow S.E. (2014). The Hygroscopic Biosurfactant Syringafactin Produced by Pseudomonas Syringae Enhances Fitness on Leaf Surfaces during Fluctuating Humidity. Environ. Microbiol..

[44] Ni Y., Guo Y.-J., Wang J., Xia R.-E., Wang X.-Q., Ash G., Li J.-N. (2014). Responses of Physiological Indexes and Leaf Epicuticular Waxes of Brassica Napus to Sclerotinia Sclerotiorum Infection. Plant Pathol..

[45] Yeats T.H., Rose J.K.C. (2013). The Formation and Function of Plant Cuticles. Plant Physiol..

[46] Wang F., Zhang P., Qiang S., Zhu Y.Z., Xu L.L. (2008). Effects of Epicuticular Wax from Digitaria Sanguinalis and Festuca Arundinacea on Infection by Curvularia Eragrostidis. Australas. Plant Pathol..

[47] Gniwotta F., Vogg G., Gartmann V., Carver T.L.W., Riederer M., Jetter R. (2005). What Do Microbes Encounter at the Plant Surface? Chemical Composition of Pea Leaf Cuticular Waxes 1. Plant Physiol..

[48] Uppalapati S.R., Ishiga Y., Doraiswamy V., Bedair M., Mittal S., Chen J., Nakashima J., Tang Y., Tadege M., Ratet P. (2012). Loss of Abaxial Leaf Epicuticular Wax in Medicago Truncatula Irg1/Palm1 Mutants Results in Reduced Spore Differentiation of Anthracnose and Nonhost Rust Pathogens. Plant Cell.

[49] Reisige K., Gorzelanny C., Daniels U., Moerschbacher B.M. (2006). The C28 Aldehyde Octacosanal Is a Morphogenetically Active Component Involved in Host Plant Recognition and Infection Structure Differentiation in the Wheat Stem Rust Fungus. Physiol. Mol. Plant Pathol..

[50] Wang W., Liu X., Gai X., Ren J., Liu X., Cai Y., Wang Q., Ren H. (2015). *Cucumis sativus* L. WAX2 Plays a Pivotal Role in Wax Biosynthesis, Influencing Pollen Fertility and Plant Biotic and Abiotic Stress Responses. Plant Cell Physiol..

[51] Podila G.K., Rogers L.M., Kolattukudy P.E. (1993). Chemical Signals from Avocado Surface Wax Trigger Germination and Appressorium Formation in Colletotrichum Gloeosporioides. Plant Physiol..

[52] Bourdenx B., Bernard A., Domergue F., Pascal S., Léger A., Roby D., Pervent M., Vile D., Haslam R.P., Napier J.A. (2011). Overexpression of Arabidopsis ECERIFERUM1 Promotes Wax Very-Long-Chain Alkane Biosynthesis and Influences Plant Response to Biotic and Abiotic Stresses. Plant Physiol..

[53] Lewandowska M., Keyl A., Feussner I. (2020). Wax Biosynthesis in Response to Danger: Its Regulation upon Abiotic and Biotic Stress. New Phytol..

[54] Raffaele S., Vailleau F., Léger A., Joubès J., Miersch O., Huard C., Blée E., Mongrand S., Domergue F., Roby D. (2008). A MYB Transcription Factor Regulates Very-Long-Chain Fatty Acid Biosynthesis for Activation of the Hypersensitive Cell Death Response in Arabidopsis. Plant Cell.

[55] Zhang Y.-L., Zhang C.-L., Wang G.-L., Wang Y.-X., Qi C.-H., Zhao Q., You C.-X., Li Y.-Y., Hao Y.-J. (2019). The R2R3 MYB Transcription Factor MdMYB30 Modulates Plant Resistance against Pathogens by Regulating Cuticular Wax Biosynthesis. BMC Plant Biol..

[56] Serrano M., Coluccia F., Torres M., L’Haridon F., Métraux J.P. (2014). The Cuticle and Plant Defense to Pathogens. Front. Plant Sci..

[57] L’Haridon F., Besson-Bard A., Binda M., Serrano M., Abou-Mansour E., Balet F., Schoonbeek H.J., Hess S., Mir R., Léon J. (2011). A Permeable Cuticle Is Associated with the Release of Reactive Oxygen Species and Induction of Innate Immunity. PLoS Pathog..

[58] Xia Y., Gao Q.M., Yu K., Lapchyk L., Navarre D.R., Hildebrand D., Kachroo A., Kachroo P. (2009). An Intact Cuticle in Distal Tissues Is Essential for the Induction of Systemic Acquired Resistance in Plants. Cell Host Microbe.

[59] Li Y., Beisson F., Koo A.J.K., Molina I., Pollard M., Ohlrogge J. (2007). Identification of Acyltransferases Required for Cutin Biosynthesis and Production of Cutin with Suberin-like Monomers. Proc. Natl. Acad. Sci. USA.

[60] Arya G.C., Cohen H. (2022). The Multifaceted Roles of Fungal Cutinases during Infection. J. Fungi.

[61] Wang Y., Chen J., Li D.W., Zheng L., Huang J. (2017). CglCUT1 Gene Required for Cutinase Activity and Pathogenicity of Colletotrichum Gloeosporioides Causing Anthracnose of Camellia Oleifera. Eur. J. Plant Pathol..

[62] Li D., Ashby A.M., Johnstone K. (2003). Molecular Evidence That the Extracellular Cutinase Pbc1 Is Required for Pathogenicity of Pyrenopeziza Brassicae on Oilseed Rape. Mol. Plant-Microbe Interact..

[63] Lu L., Rong W., Massart S., Zhang Z. (2018). Genome-Wide Identification and Expression Analysis of Cutinase Gene Family in Rhizoctonia Cerealis and Functional Study of an Active Cutinase RcCUT1 in the Fungal-Wheat Interaction. Front. Microbiol..

[64] Liu T., Hou J., Wang Y., Jin Y., Borth W., Zhao F., Liu Z., Hu J., Zuo Y. (2016). Genome-Wide Identification, Classification and Expression Analysis in Fungal–Plant Interactions of Cutinase Gene Family and Functional Analysis of a Putative ClCUT7 in Curvularia Lunata. Mol. Genet. Genom..

[65] Leroch M., Kleber A., Silva E., Coenen T., Koppenhöfer D., Shmaryahu A., Valenzuela P.D.T., Hahn M. (2013). Transcriptome Profiling of Botrytis Cinerea Conidial Germination Reveals Upregulation of Infection-Related Genes during the Prepenetration Stage. Eucaryotic Cell.

[66] Gui Y.J., Zhang W.Q., Zhang D.D., Zhou L., Short D.P.G., Wang J., Ma X.F., Li T.G., Kong Z.Q., Wang B.L. (2018). A Verticillium Dahliae Extracellular Cutinase Modulates Plant Immune Responses. Mol. Plant-Microbe Interact..

[67] Stahl D.J., Schäfer W. (1992). Cutinase Is Not Required for Fungal Pathogenicity on Pea. Plant Cell.

[68] Sweigard J.A., Chumley F.G., Valent B. (1992). Disruption of a Magnaporthe Grisea Cutinase Gene. Mol. Gen. Genet..

[69] Li D., Sirakova T., Rogers L., Ettinger W.F., Kolattukudy P.E. (2002). Regulation of Constitutively Expressed and Induced Cutinase Genes by Different Zinc Finger Transcription Factors in *Fusarium solani* f. Sp. Pisi (*Nectria haematococca*). J. Biol. Chem..

[70] Martins I., Hartmann D.O., Alves P.C., Martins C., Garcia H., Leclercq C.C., Ferreira R., He J., Renaut J., Becker J.D. (2014). Elucidating How the Saprophytic Fungus Aspergillus Nidulans Uses the Plant Polyester Suberin as Carbon Source. BMC Genomics.

[71] Xiao F., Goodwin S.M., Xiao Y., Sun Z., Baker D., Tang X., Jenks M.A., Zhou J.M. (2004). Arabidopsis CYP86A2 Represses Pseudomonas Syringae Type III Genes and Is Required for Cuticle Development. EMBO J..

[72] Fauth M., Schweizer P., Buchala A., Markstadter C., Riederer M., Kato T., Kauss H. (1998). Cutin Monomers and Surface Wax Constituents Elicit H2O2 in Conditioned Cucumber Hypocotyl Segments and Enhance the Activity of Other H2O2 Elicitors. Plant Physiol..

[73] Schweizer P., Jeanguenat A., Whitacre D., Métraux J.P., Mösinger E. (1996). Induction of Resistance in Barley against Erysiphe Graminis f.Sp. Hordei by Free Cutin Monomers. Physiol. Mol. Plant Pathol..

[74] Schweizer P., Jeanguenat A., Mösinger E., Métraux J.P. (1994). Plant Protection by Free Cutin Monomers in Two Cereal Pathosystems. Adv. Mol. Genet. Plant-Microbe Interact..

[75] Tanaka K., Heil M. (2021). Damage-Associated Molecular Patterns (DAMPs) in Plant Innate Immunity: Applying the Danger Model and Evolutionary Perspectives. Annu. Rev. Phytopathol..

[76] Zhao J. (2015). Phospholipase D and Phosphatidic Acid in Plant Defence Response: From Protein-Protein and Lipid-Protein Interactions to Hormone Signalling. J. Exp. Bot..

[77] Casillas-Vargas G., Ocasio-Malavé C., Medina S., Morales-Guzmán C., Del Valle R.G., Carballeira N.M., Sanabria-Ríos D.J. (2021). Antibacterial Fatty Acids: An Update of Possible Mechanisms of Action and Implications in the Development of the next-Generation of Antibacterial Agents. Prog. Lipid Res..

[78] McGaw L.J., Jäger A.K., Van Staden J. (2002). Antibacterial Effects of Fatty Acids and Related Compounds from Plants. S. Afr. J. Bot..

[79] Liu S., Ruan W., Li J., Xu H., Wang J., Gao Y., Wang J. (2008). Biological Control of Phytopathogenic Fungi by Fatty Acids. Mycopathologia.

[80] Walters D., Raynor L., Mitchell A., Walker R., Walker K. (2004). Antifungal Activities of Four Fatty Acids against Plant Pathogenic Fungi. Mycopathologia.

[81] Guimarães A., Venâncio A. (2022). The Potential of Fatty Acids and Their Derivatives as Antifungal Agents: A Review. Toxins.

[82] Yaeno T., Matsuda O., Iba K. (2004). Role of Chloroplast Trienoic Fatty Acids in Plant Disease Defense Responses. Plant J..

[83] Xiao R., Zou Y., Guo X., Li H., Lu H. (2022). Fatty Acid Desaturases (FADs) Modulate Multiple Lipid Metabolism Pathways to Improve Plant Resistance. Mol. Biol. Rep..

[84] Nandi A., Moeder W., Kachroo P., Klessig D.F., Shah J. (2005). Arabidopsis Ssi2-Conferred Susceptibility to Botrytis Cinerea Is Dependent on EDS5 and PAD4. Mol. Plant-Microbe Interact..

[85] Chandra-Shekara A.C., Venugopal S.C., Barman S.R., Kachroo A., Kachroo P. (2007). Plastidial Fatty Acid Levels Regulate Resistance Gene-Dependent Defense Signaling in Arabidopsis. Proc. Natl. Acad. Sci. USA.

[86] Upchurch R.G. (2008). Fatty Acid Unsaturation, Mobilization, and Regulation in the Response of Plants to Stress. Biotechnol. Lett..

[87] Ruan J., Zhou Y., Zhou M., Yan J., Khurshid M., Weng W., Cheng J., Zhang K. (2019). Jasmonic Acid Signaling Pathway in Plants. Int. J. Mol. Sci..

[88] Blée E. (2002). Impact of Phyto-Oxylipins in Plant Defense. Trends Plant Sci..

[89] Mosblech A., Feussner I., Heilmann I. (2009). Oxylipins: Structurally Diverse Metabolites from Fatty Acid Oxidation. Plant Physiol. Biochem..

[90] Wang Y., Mostafa S., Zeng W., Jin B. (2021). Function and Mechanism of Jasmonic Acid in Plant Responses to Abiotic and Biotic Stresses. Int. J. Mol. Sci..

[91] Robert-Seilaniantz A., Grant M., Jones J.D.G. (2011). Hormone Crosstalk in Plant Disease and Defense: More Than Just JASMONATE-SALICYLATE Antagonism. Annu. Rev. Phytopathol..

[92] Berens M.L., Berry H.M., Mine A., Argueso C.T., Tsuda K. (2017). Evolution of Hormone Signaling Networks in Plant Defense. Annu. Rev. Phytopathol..

[93] Macioszek V.K., Jęcz T., Ciereszko I., Kononowicz A.K. (2023). Jasmonic Acid as a Mediator in Plant Response to Necrotrophic Fungi. Cells.

[94] Deboever E., Deleu M., Mongrand S., Lins L., Fauconnier M.-L. (2020). Plant-Pathogen Interactions: Underestimated Roles of Phyto-Oxylipins. Trends Plant Sci..

[95] Li M., Yu G., Cao C., Liu P. (2021). Metabolism, Signaling, and Transport of Jasmonates. Plant Commun..

[96] Ghorbel M., Brini F., Sharma A., Landi M. (2021). Role of Jasmonic Acid in Plants: The Molecular Point of View. Plant Cell Rep..

[97] Jimenez Aleman G.H., Thirumalaikumar V.P., Jander G., Fernie A.R., Skirycz A. (2022). OPDA, More than Just a Jasmonate Precursor. Phytochemistry.

[98] Scalschi L., Sanmartín M., Camañes G., Troncho P., Sánchez-Serrano J.J., García-Agustín P., Vicedo B. (2015). Silencing of OPR3 in Tomato Reveals the Role of OPDA in Callose Deposition during the Activation of Defense Responses against Botrytis Cinerea. Plant J..

[99] Shinya T., Miyamoto K., Uchida K., Hojo Y., Yumoto E., Okada K., Yamane H., Galis I. (2022). Chitooligosaccharide Elicitor and Oxylipins Synergistically Elevate Phytoalexin Production in Rice. Plant Mol. Biol..

[100] Taki N., Sasaki-Sekimoto Y., Obayashi T., Kikuta A., Kobayashi K., Ainai T., Yagi K., Sakurai N., Suzuki H., Masuda T. (2005). 12-Oxo-Phytodienoic Acid Triggers Expression of a Distinct Set of Genes and Plays a Role in Wound-Induced Gene Expression in Arabidopsis. Plant Physiol..

[101] Wang K.D., Borrego E.J., Kenerley C.M., Kolomiets M.V. (2020). Oxylipins Other Than Jasmonic Acid Are Xylem-Resident Signals Regulating Systemic Resistance Induced by Trichoderma Virens in Maize. Plant Cell.

[102] Knieper M., Viehhauser A., Dietz K.J. (2023). Oxylipins and Reactive Carbonyls as Regulators of the Plant Redox and Reactive Oxygen Species Network under Stress. Antioxidants.

[103] Wasternack C., Hause B. (2016). OPDA-Ile—A New JA-Ile-Independent Signal?. Plant Signal. Behav..

[104] Mueller S., Hilbert B., Dueckershoff K., Roitsch T., Krischke M., Mueller M.J., Berger S., Biowissenschaften J., Biologie P., Wuerzburg U. (2008). General Detoxification and Stress Responses Are Mediated by Oxidized Lipids through TGA Transcription Factors in Arabidopsis. Plant Cell.

[105] Kourtchenko O., Andersson M.X., Hamberg M., Brunnström Å., Göbel C., McPhail K.L., Gerwick W.H., Feussner I., Ellerström M. (2007). Oxo-Phytodienoic Acid-Containing Galactolipids in Arabidopsis: Jasmonate Signaling Dependence. Plant Physiol..

[106] Nilsson A.K., Johansson O.N., Fahlberg P., Steinhart F., Gustavsson M.B., Ellerström M., Andersson M.X. (2014). Formation of Oxidized Phosphatidylinositol and 12-Oxo-Phytodienoic Acid Containing Acylated Phosphatidylglycerol during the Hypersensitive Response in Arabidopsis. Phytochemistry.

[107] Liu W., Park S.-W. (2021). 12-Oxo-Phytodienoic Acid: A Fuse and/or Switch of Plant Growth and Defense Responses?. Front. Plant Sci..

[108] Cheong H., Dos Santos I.B., Liu W., Gosse H.N., Park S.-W., Liu W., Gosse H.N., Park W. (2017). Cyclophilin 20-3 Is Positioned as a Regulatory Hub between Light-Dependent Redox and 12-Oxo-Phytodienoic Acid Signaling. Plant Signal. Behav..

[109] Gorman Z., Tolley J.P., Koiwa H., Kolomiets M.V. (2021). The Synthesis of Pentyl Leaf Volatiles and Their Role in Resistance to Anthracnose Leaf Blight. Front. Plant Sci..

[110] Tolley J.P., Gorman Z., Lei J., Yeo I.-C., Nagashima Y., Jashi V., Zhu-Salzman K., Kolomiets M.V., Koiwa H. (2023). Overexpression of Maize ZmLOX6 in Arabidopsis Thaliana Enhances Damage-Induced Pentyl Leaf Volatile Emissions That Affect Plant Growth and Interaction with Aphids. J. Exp. Bot..

[111] Gorman Z., Christensen S.A., Yan Y., He Y., Borrego E., Kolomiets M.V. (2020). Green Leaf Volatiles and Jasmonic Acid Enhance Susceptibility to Anthracnose Diseases Caused by Colletotrichum Graminicola in Maize. Mol. Plant Pathol..

[112] Song G.C., Ryu C.M. (2013). Two Volatile Organic Compounds Trigger Plant Self-Defense against a Bacterial Pathogen and a Sucking Insect in Cucumber under Open Field Conditions. Int. J. Mol. Sci..

[113] Song G.C., Choi H.K., Ryu C.-M. (2015). Gaseous 3-Pentanol Primes Plant Immunity against a Bacterial Speck Pathogen, Pseudomonas Syringae Pv. Tomato via Salicylic Acid and Jasmonic Acid-Dependent Signaling Pathways in Arabidopsis. Front. Plant Sci..

[114] Shen J., Tieman D., Jones J.B., Taylor M.G., Schmelz E., Huffaker A., Bies D., Chen K., Klee H.J. (2014). A 13-Lipoxygenase, TomloxC, Is Essential for Synthesis of C5 Flavour Volatiles in Tomato. J. Exp. Bot..

[115] Vicente J., Cascón T., Vicedo B., García-Agustín P., Hamberg M., Castresana C. (2012). Role of 9-Lipoxygenase and α-Dioxygenase Oxylipin Pathways as Modulators of Local and Systemic Defense. Mol. Plant.

[116] Göbel C., Feussner I., Schmidt A., Scheel D., Sanchez-Serrano J., Hamberg M., Rosahl S. (2001). Oxylipin Profiling Reveals the Preferential Stimulation of the 9-Lipoxygenase Pathway in Elicitor-Treated Potato Cells. J. Biol. Chem..

[117] Mène-Saffrané L., Esquerré-Tugayé M.T., Fournier J. (2003). Constitutive Expression of an Inducible Lipoxygenase in Transgenic Tobacco Decreases Susceptibility to Phytophthora Parasitica Var. Nicotianae. Mol. Breed..

[118] Fauconnier M.-L., Rojas-Beltran J., Dupuis B., Delaplace P., Frettinger P., Gosset V., du Jardin P. (2008). Changes in Oxylipin Synthesis after Phytophthora Infestans Infection of Potato Leaves Do Not Correlate with Resistance. Plant Physiol. Biochem..

[119] Zoeller M., Stingl N., Krischke M., Fekete A., Waller F., Berger S., Mueller M.J. (2012). Lipid Profiling of the Arabidopsis Hypersensitive Response Reveals Specific Lipid Peroxidation and Fragmentation Processes: Biogenesis of Pimelic and Azelaic Acid. Plant Physiol..

[120] Lim G.H., Singhal R., Kachroo A., Kachroo P. (2017). Fatty Acid- and Lipid-Mediated Signaling in Plant Defense. Annu. Rev. Phytopathol..

[121] Gao Q.M., Yu K., Xia Y., Shine M.B., Wang C., Navarre D.R., Kachroo A., Kachroo P. (2014). Mono- and Digalactosyldiacylglycerol Lipids Function Nonredundantly to Regulate Systemic Acquired Resistance in Plants. Cell Rep..

[122] Thoma I., Loeffler C., Sinha A.K., Gupta M., Krischke M., Steffan B., Roitsch T., Mueller M.J. (2003). Cyclopentenone Isoprostanes Induced by Reactive Oxygen Species Trigger Defense Gene Activation and Phytoalexin Accumulation in Plants. Plant J..

[123] Loeffler C., Berger S., Guy A., Durand T., Bringmann G., Mueller M.J., Dreyer M., Von Rad U., Durner J., Mueller M.J. (2005). B1-Phytoprostanes Trigger Plant Defense and Detoxification Responses. Plant Physiol..

[124] Christensen S.A., Kolomiets M.V. (2011). The Lipid Language of Plant—Fungal Interactions. Fungal Genet. Biol..

[125] Tsitsigiannis D.I., Kunze S., Willis D.K., Feussner I., Keller N.P. (2005). Aspergillus Infection Inhibits the Expression of Peanut 13S-HPODE-Forming Seed Lipoxygenases. Mol. Plant-Microbe Interact..

[126] Brodhagen M., Tsitsigiannis D.I., Hornung E., Goebel C., Feussner I., Keller N.P. (2008). Reciprocal Oxylipin-Mediated Cross-Talk in the Aspergillus—Seed Pathosystem. Mol. Microbiol..

[127] Beccaccioli M., Reverberi M., Scala V. (2019). Fungal Lipids: Biosynthesis and Signalling during Plant-Pathogen Interaction. Front. Biosci. Landmark.

[128] Battilani P., Lanubile A., Scala V., Reverberi M., Gregori R., Falavigna C., Dall’Asta C., Park Y.-S., Bennett J., Borrego E.J. (2018). Oxylipins from Both Pathogen and Host Antagonize Jasmonic Acid-Mediated Defence via the 9-Lipoxygenase Pathway in Fusarium Verticillioides Infection of Maize. Mol. Plant Pathol..

[129] Eckardt N.A. (2008). Oxylipin Signaling in Plant Stress Responses. Plant Cell.

[130] Andersson M.X., Hamberg M., Kourtchenko O., Brunnstro Å., McPhail K.L., Gerwick W.H., Göbel C., Feussner I., Ellerström M. (2006). Oxylipin Profiling of the Hypersensitive Response in Arabidopsis Thaliana: Formation of a Novel Oxo-Phytodienoic Acid-Containing Galactolipid, Arabidopside E. J. Biol. Chem..

[131] Han Z., Xiong D., Schneiter R., Tian C. (2023). The Function of Plant PR1 and Other Members of the CAP Protein Superfamily in Plant–Pathogen Interactions. Mol. Plant Pathol..

[132] Safi H., Saibi W., Alaoui M.M., Hmyene A., Masmoudi K., Hanin M., Brini F. (2015). A Wheat Lipid Transfer Protein (TdLTP4) Promotes Tolerance to Abiotic and Biotic Stress in Arabidopsis Thaliana. Plant Physiol. Biochem..

[133] Patkar R.N., Chattoo B.B. (2006). Transgenic Indica Rice Expressing Ns-LTP-like Protein Shows Enhanced Resistance to Both Fungal and Bacterial Pathogens. Mol. Breed..

[134] Gao S., Guo W., Feng W., Liu L., Song X., Chen J., Hou W., Zhu H., Tang S., Hu J. (2016). LTP3 Contributes to Disease Susceptibility in Arabidopsis by Enhancing Abscisic Acid (ABA) Biosynthesis. Mol. Plant Pathol..

[135] Buhot N., Gomès E., Milat M.-L., Ponchet M., Marion D., Lequeu J., Delrot S., Coutos-Thévenot P., Blein J.-P. (2004). Modulation of the Biological Activity of a Tobacco LTP1 by Lipid Complexation. Mol. Biol. Cell.

[136] Girault T., François J., Rogniaux H., Pascal S., Delrot S., Coutos-Thévenot P., Gomès E. (2008). Exogenous Application of a Lipid Transfer Protein-Jasmonic Acid Complex Induces Protection of Grapevine towards Infection by Botrytis Cinerea. Plant Physiol. Biochem..

[137] Gao H., Ma K., Ji G., Pan L., Zhou Q. (2022). Lipid Transfer Proteins Involved in Plant—Pathogen Interactions and Their Molecular Mechanisms. Mol. Plant Pathol..

[138] Jung H.W., Tschaplinski T.J., Wang L., Glazebrook J., Greenberg J.T. (2009). Priming in Systemic Resistance. Science (80-.).

[139] Zeng H.Y., Bao H.N., Chen Y.L., Chen D.K., Zhang K., Liu S.K., Yang L., Li Y.K., Yao N. (2022). The Two Classes of Ceramide Synthases Play Different Roles in Plant Immunity and Cell Death. Front. Plant Sci..

[140] Berkey R., Bendigeri D., Xiao S. (2012). Sphingolipids and Plant Defense/Disease: The “Death” Connection and Beyond. Front. Plant Sci..

[141] Kong L., Ma X., Zhang C., Kim S.-I., Li B., Xie Y., Yeo I.-C., Thapa H., Chen S., Devarenne T.P. (2024). Dual Phosphorylation of DGK5-Mediated PA Burst Regulates ROS in Plant Immunity. Cell.

[142] De Jong C.F., Laxalt A.M., Bargmann B.O.R., De Wit P.J.G.M., Joosten M.H.A.J., Munnik T. (2004). Phosphatidic Acid Accumulation Is an Early Response in the Cf-4/Avr4 Interaction. Plant J..

[143] Li J., Wang X. (2019). Phospholipase D and Phosphatidic Acid in Plant Immunity. Plant Sci..

[144] Xing J., Zhang L., Duan Z., Lin J. (2021). Coordination of Phospholipid-Based Signaling and Membrane Trafficking in Plant Immunity. Trends Plant Sci..

[145] Pečenková T., Pleskot R., Žárský V. (2017). Subcellular Localization of Arabidopsis Pathogenesis-Related 1 (PR1) Protein. Int. J. Mol. Sci..

[146] Xing J., Li X., Wang X., Lv X., Wang L., Zhang L., Zhu Y., Shen Q., Baluška F., Šamaj J. (2019). Secretion of Phospholipase Dδ Functions as a Regulatory Mechanism in Plant Innate Immunity. Plant Cell.

[147] Pinosa F., Buhot N., Kwaaitaal M., Fahlberg P., Thordal-Christensen H., Ellerström M., Andersson M.X. (2013). Arabidopsis Phospholipase Dδ Is Involved in Basal Defense and Nonhost Resistance to Powdery Mildew Fungi. Plant Physiol..

